# The Editor's Letter-Box

**Published:** 1920-07-10

**Authors:** A. E. Collins

**Affiliations:** Secretary. Staffordshire General Infirmary, Stafford.


**The Editor's Letter Box—(*continued*).**

### "The Story of Hospital Sunday."

*To the Editor of* The Hospital.

*To the Editor of* The Hospital.

Sir,—As a young hospital secretary and a hospital worker for many years, I delight to read your valuable paper each week. In your issue of June 26 last there appears an article, "The Story of Hospital Sunday." The Staffordshire General Infirmary cannot, as far as I am aware, record the fact of a Hospital Sunday collection as far back as 1792, but a charity sermon was preached throughout the diocese in 1801 for the benefit of the institution, and resulted in the sum of £1,364 3s. 8d. being handed to the charity. In 1861 Hospital Sunday was established in Stafford, and ever since that year contributions have been received from the Hospital Sunday Committee. The Rev. J. Henn, the Secretary of the Hospital Sunday movement in Manchester, in the letter to the late Sir Henry Burdett referred to in your article, is a little out of it when he states that all the rest of the hospitals are copies of theirs, which was inaugurated in 1870. Stafford can claim at least nine years start.—Yours faithfully, A. E. Collins, Secretary.

Staffordshire General Infirmary, Stafford.

